# Examining the Myth of Prescribed Stimulant Misuse among Individuals with Attention-Deficit/Hyperactivity Disorder: A Systematic Review

**DOI:** 10.3390/ph17081076

**Published:** 2024-08-16

**Authors:** Tommaso Callovini, Delfina Janiri, Daniele Segatori, Giulia Mastroeni, Georgios D. Kotzalidis, Marco Di Nicola, Gabriele Sani

**Affiliations:** 1 Department of Neuroscience, Section of Psychiatry, Università Cattolica del Sacro Cuore, 00168 Rome, Italydottorsegatori@gmail.com (D.S.); giorgio.kotzalidis@gmail.com (G.D.K.);; 2 Department of Psychiatry, Section of Psychiatry, Fondazione Policlinico Universitario Agostino Gemelli IRCCS, 00168 Rome, Italy

**Keywords:** ADHD, prescribed stimulant, psychostimulant misuse, methylphenidate, amphetamine

## Abstract

The literature emphasizes the importance of addressing the misuse of ADHD medications as a potential significant healthcare issue within the general population. Nevertheless, there are no systematic reviews that specifically examine whether the misuse of psychostimulant medication among clinical populations diagnosed with ADHD who are undergoing prescribed stimulant therapy is a rational concern or a false myth. This systematic review was carried out according to the Preferred Reporting Items for Systematic Reviews and Meta-Analyses (PRISMA) 2020 Statement. We searched PubMed databases for articles indexed up to 12th July 2023, without language restrictions. Our systematic search generated 996 unique articles. After a full-text revision, 13 studies met the eligibility criteria and were included in the systematic review. In the 50% of the study on the adult population, the reported prevalence of stimulant misuse was 0%. In other studies, the range of stimulant misuse rates varied from 2% to 29%, with no available data specifically focusing on the youth population. It has been noted that misuse of prescribed stimulant treatment is linked with particular subject characteristics, such as older age, prior or more frequent use of ADHD medication, use of short-acting medication, and a history of alcohol/substance misuse diagnosis. Despite certain limitations, our study highlights that while a significant proportion of individuals undergoing psychostimulant treatment for ADHD follow their prescribed medication regimens without resorting to misuse behaviors, there is variability in adherence, with occurrences of misuse behaviors. The misuse of prescribed ADHD treatment appears to be associated with distinct subject characteristics, underscoring the importance for tailored interventions addressing the specific requirements of these individuals to attain optimal treatment outcomes while mitigating misuse risks.

## 1. Introduction

Attention Deficit Hyperactivity Disorder (ADHD) stands as one of the most prevalent psychiatric disorders among children and adolescents, with an estimated prevalence rate of 3–5% [1]. Extensive evidence indicate that ADHD is not merely confined to childhood but often persists across the lifespan, affecting approximately 2.5% of the global adult population [2]. Recent years have seen a discernible rise in the prescription of stimulants in both adult and pediatric populations with regard to available treatments [3,4]. The widespread adoption of psychostimulant medications presents a dual narrative. On one hand, these medications, including methylphenidate and amphetamines, are considered the first-line pharmacological treatment for ADHD as they exert moderate-to-high clinical effects, with average effects higher than atomoxetine and other non-stimulants [5].

On the other hand, there is growing concern about the potential for misuse and diversion of these drugs. Prescribed psychostimulants have been the center of controversy due to their abuse potential, which is linked to their mechanism of action of increasing norepinephrine and dopamine signaling in the brain [3,6]. The literature underscores the significance of misuse (taking the medication without following medical instructions) and diversion (selling or giving away prescribed medication) of ADHD medications as critical healthcare concerns among the general population, particularly within college campuses. A recent meta-analysis of 30 studies revealed that 17% of college students have engaged in stimulant medication misuse, with the main motivation reported being cognitive and academic enhancement [7]. Interestingly, symptoms of inattention [8,9], impulsivity and internal restlessness [10,11,12] were reported among the risk factors predictive of prescription stimulant misuse. Metanalytic evidence suggested that ADHD symptoms were significantly associated with prescription stimulant misuse, possibly indicating that this concerning trend may affect individuals with undiagnosed ADHD and not undergoing appropriate stimulant therapy [7]. Conversely to date, a systematic review focusing on misuse and diversion of psychostimulant medication among clinical populations of individuals diagnosed with ADHD undergoing prescribed stimulant therapy is still lacking. To bridge this gap, we conducted a systematic review to elucidate the prevalence of misuse and diversion of regularly prescribed psychostimulant medications among patients with ADHD. Our goal is to shed light on the potential abuse of these medications and promote their appropriate use. 

## 2. Results

**Figure 1:**
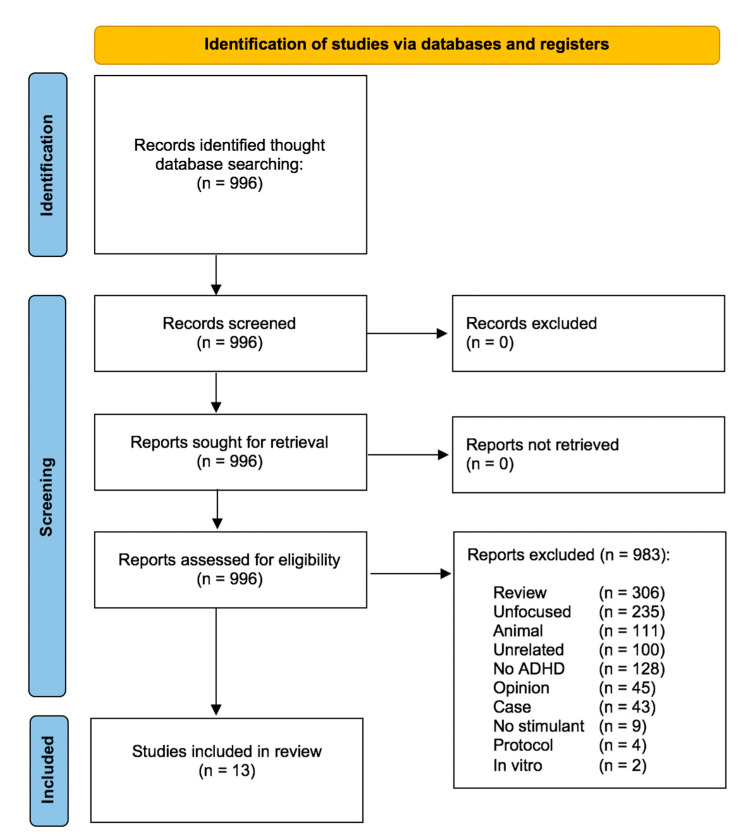
Flowchart of the inclusion process.

The study selection process is described in Figure 1. Our systematic search generated 996 unique articles. After a full-text revision, 13 studies met the eligibility criteria and were included in the systematic review. We included one double-blind controlled trial [13], two open label trials [14,15], five cross-sectional observational studies [16,17,18,19,20], and five longitudinal observational studies [21,22,23,24,25]. Eight studies were conducted on an adult sample [13,14,15,17,19,21,22,23], two on a youth sample [16,17,18,19,20], and three on a mixed adult and youth sample [18,24,25]. A summary of the included articles is displayed in Table 1 and Table S1.

Overall, 11 studies reported rates of misuse, and 4 studies reported rates of diversion. The reported information about the misuse or diversion was not totally regarding the index population (individuals with ADHD undergoing a stimulant therapy) in five studies [16,18,19,23,24]. In three studies [16,19,23], 54.5%, 29.3%, and 5.03%, respectively, of the samples were not undergoing stimulant therapy. In one study [18], 10.8% of the sample did not have an ADHD diagnosis or was not undergoing stimulant therapy. In one study [24], 22.5% of the sample did not have an ADHD diagnosis. Among the studies focusing on the adult population, four reported no evidence of stimulant misuse [13,14,15,22], four reported a rate of misuse ranging from 8.6 % to 29 % [17,19,21,23], and three reported a rate of diversion ranging from 0% to 44% [13,17,21].

In the studies concerning the youth population, one study conducted on subjects with ADHD–SUD (Substance Use Disorder) comorbidity reported a high percentage of lifetime psychostimulant misuse (41.8%); however, the prevalence of abusers among our target population, that is individuals with current ADHD and prescribed psychostimulant medication, is not specified [16]. The authors did not distinguish between patients who were taking medications and those who were not. Moreover, the study reported that 20% of the patients attested diverted psychostimulant medication [16]. The other selected study that analyzed the young population reported a diversion rate of 1%, without reporting data on stimulant misuse [20].

In research involving both adults and youth, three reported the stimulant misuse rate ranging from 2.1% to 14.3% [18,24,25] and one reported that 16.5% of the sample diverted ADHD medication [18].

Four studies reported characteristics associated with stimulant misuse or diversion [17,19,24,25]. In particular, misuse is reported to be associated with older age [19,24,25], more frequent psychoactive drugs use (benzodiazepine, morphine, opiate substitution treatment) [25], more frequent use of immediate-release methylphenidate (Ritalin) and less extended release (Concerta) [25], previous diagnosis of alcohol and drug misuse [17,21,24], fewer positive and negative expectancies [19], and previous or more frequent use of ADHD medication [19,24].

## 3. Discussion

Based on data from the 13 included studies, our systematic review identified varying rates of misuse and diversion among clinical samples of individuals undergoing psychostimulant therapy for ADHD across different age groups. The prevalence of stimulant misuse presents heterogenous results. Among adults, half of the included studies reported no evidence of stimulant misuse [13,14,15,22]. This finding may indicate that stimulant misuse is not universally prevalent among adults undergoing ADHD therapy. This observation is encouraging and underscores the potential effectiveness of appropriate clinical management and adherence to treatment protocols among this population [26]. This clinical observation is consistent with preclinical evidence showing that oral administration of clinical doses of stimulants can increase extracellular norepinephrine concentrations in the hippocampus without significantly affecting extracellular dopamine in the nucleus accumbens [27], which is known to be responsible for addictive behaviors [28]. The notion of a low potential for abuse is further supported by the clinical evidence of a high discontinuation rate of stimulant treatment for ADHD, reported to be up to 65% [29]. Conversely, the other half of included studies with the adult sample reported evidence of stimulant misuse, with rates ranging from 8.6% to 29% [17,19,21,23]. Nevertheless, these studies show highly variable rates of abuse, possibly because they have small samples and heterogeneous methods for assessing abuse. In particular, considering study designs, we note that the two cross-sectional studies [17,19] reported higher prevalence of abuse (approximately 30%) compared to the longitudinal studies [13,14,15,21,22,23], which reported abuse in only two studies, ranging from 8.6% [23] to 22% [21]. It is important to underline that the reported prevalence of abuse seems to also be influenced by the duration of the study, considering that all the studies reporting no evidence of abuse during the study period ranged from 8 weeks to 2 years [13,14,15,22], compared to [23] and [21], which reported a study period ranging from 4.5 to 10 years. Moreover, in one study, the reported absence of stimulant misuse [15] is probably confounded by the reduced opportunities for abuse in a prison setting. The prevalence of misuse among larger sample of mixed youth and adult populations appears lower, ranging from 2.1% to 14.3% [18,24,25]. The considerations regarding the study design are similar to those for the adult sample, with the cross-sectional study reporting a higher rate of abuse at 14.3% [18] compared to the rates reported from the two longitudinal studies, namely 8.42% [24] and 2.1% [25]. Data on misuse available in the purely youth population are limited to only one study conducted on youths, which reported a 41.8% abuse rate; however, the prevalence of abusers among our target population, that is individuals with current ADHD and prescribed psychostimulant medication, is not specified [16]. According to our results, several factors emerge as potential contributors to stimulant misuse among individuals with ADHD. Notably, older age [19,24,25] and previous or more frequent use of ADHD medication [19,24] were consistently associated with higher rates of misuse.

These correlations may stem from the widespread phenomenon of tolerance to stimulant pharmacotherapy, which is recognized to be dose-dependent and increasing over time [30]. Interestingly, there are differences in the age of misuse between the general non-clinical population and our clinical ADHD sample. Specifically, while our review found that older age is associated with higher rates of misuse among individuals with ADHD, other studies not included in our review indicate that misuse is more prevalent among young adults aged 18–25 in the general population [31]. This suggests different patterns of misuse between the general population and individuals with ADHD, possibly pointing to misuse being secondary to drug tolerance in the latter population. Our results also highlighted that misuse was associated with more frequent use of immediate-release methylphenidate, while extended release formulation was associated with the non-misuser’s group [25]. Indeed, the rate of rise of a drug’s plasma concentration has been thought to indicate abuse liability of different drugs, including methylphenidate [32]. With extended-release stimulants, the slower rise and fall of stimulant levels in the brain may contribute to decreased drug abuse potential, yielding significantly lower drug likeability ratings compared with the immediate release formulation, regardless of peak plasma levels and dopamine transporter occupancy [33]. Additionally, co-occurring alcohol/substance use emerged as significant risk factors for prescribed stimulant misuse [17,21,24], as well as the concomitant use of psychoactive drugs (benzodiazepine, morphine, opiate substitution treatment) [25]. This pattern of poly-substance use suggests that individuals who misuse prescription stimulants are often involved in broader substance-use behaviors, which may be part of a general tendency towards risk-taking and sensation-seeking [34]. However, it is also suggested that individuals with ADHD and co-morbid SUD need higher stimulant doses to achieve optimal ADHD symptom control considering that these individuals might have developed a tolerance to central stimulants [25]. An increase in tolerance may lead to a necessity for higher doses than prescribed, potentially categorizing the subject as a misuser [35]. This is particularly significant considering the absence of clear treatment guidelines, as clinicians may hesitate to escalate doses to optimal levels for individuals with ADHD and SUD [36,37].

Interventions should be context-specific; several problems are encountered when dealing with jailed populations, where ADHD persons abound, with heterogeneity of approaches emerging and recommendations varying [38]. Training programs for primary care providers may help collaboration among physicians, staff, and pharmacists and reduce psychostimulant diversion in students with ADHD [39]. Given the high co-morbidity between ADHD, SUD, and mood disorders, special attention should be paid to addressing these conditions simultaneously to improve outcomes [40,41]. Conjoint efforts of all authorities will affect policies and inform better patients with ADHD on the perils of misusing and diverting their psychostimulant medications.

Despite the insights provided by our systematic review, several limitations warrant consideration. The small sample size of individual studies and the heterogeneity of the study designs and populations (the reasons why we were unable to meta-analyze our data), particularly the limited focus on adolescent populations, may limit the generalizability and the reliability of our findings. Additionally, the dearth of data specifically targeting adolescents underscores the need for further research to elucidate patterns of stimulant misuse and diversion in this demographic. In many studies, key information regarding the dosage and the age at the start of medication is not available, making the interpretation of findings more challenging. Furthermore, data of psychostimulants were pooled and did not distinguish among the individual drugs. Future studies should adopt standardized methodologies and longitudinal designs to assess the long-term impact of stimulant therapy on misuse behaviors and associated outcomes, hopefully reporting separate data on each psychostimulant. Moreover, by incorporating qualitative approaches, future studies could provide insights into the motivations and experiences of individuals engaging in stimulant misuse or diversion, thereby elucidating the underlying mechanisms associated with specific characteristics of the subjects behind psychostimulant misuse and diversion, which are currently lacking in our review.

## 4. Materials and Methods

This systematic review was carried out according to the Preferred Reporting Items for Systematic Reviews and Meta-Analyses (PRISMA) 2020 Statement [42].

### 4.1. Eligibility Criteria

We included studies that presented prevalence data on the misuse or diversion of ADHD stimulant medication among individuals, both children and adults, diagnosed with ADHD and undergoing prescribed stimulant therapy. We also included studies that primarily focused on the general population, provided they offered separate data on stimulant misuse or diversion, specifically among individuals diagnosed with ADHD and undergoing psychostimulant therapy. If the reported misuse or diversion data were not entirely specific to the index population, we explicitly highlighted this in the results section. We excluded (i) reviews and meta-analyses; (ii) studies not providing information on misuse/abuse/dependence or diversion of prescribed stimulants; (iii) studies that did not include ADHD-diagnosed or mixed ADHD and non-ADHD populations without providing separate data; and (iv) self-reported ADHD diagnosis. We also excluded studies with incomplete data or not undergoing peer-review process, such as conference abstracts, dissertations, and gray literature.

### 4.2. Search Strategy and Selection of Studies

We searched PubMed databases for articles indexed up to 12th July 2023, without language restrictions, using the following search phrase: (misuse* OR abuse* OR “substance use disorder*” OR illicit OR voluptuary OR recreational OR diversion OR diverted OR shunting) AND (prescription* OR prescribed OR therapeutic) AND (ADHD OR “attention deficit/hyperactivity disorder” OR “minimal brain damage” OR “minimal brain dysfunction”) AND (psychostimulant* OR stimulant* OR methylphenidate OR ritalin* OR amphetamine* OR Lisdexamfetamine OR L-Lysine-d-amphetamine OR modafinil OR adrafinil OR Amfetamine* OR Dextroamphetamine* OR dexamphetamin* OR “Mixed amphetamine salt*” OR Elvanse OR Venvanse OR Adderall OR Dexedrin* OR Vyvanse OR ProCentra OR Dyanavel OR Evekeo OR Zenzedi OR Desoxyn OR Metadate OR Concerta OR Daytrana OR Ritalin OR Methylin OR Quillivant OR Focalin OR Biphentin OR Phenida* OR Inspiral OR dexmethylphenidate OR Medikinet OR Equasym OR Penid OR Rubifen OR Aptensio OR Attentin). Four authors independently completed the preliminary screening based on titles and abstracts. Full texts were then retrieved to assess studies according to inclusion criteria for final eligibility. Disagreements concerning suitability for inclusion were resolved by discussion and consensus, involving all authors.

### 4.3. Data Extraction 

Specific data from eligible full-text articles were carefully extracted and entered into the developed extraction form. The results extracted, when available from each eligible study, were as follows: (i) data on misuse (taking the medication without following medical instructions) or diversion (selling or giving away prescribed medication) of prescribed stimulants medication; (ii) demographic and clinical characteristics of included patients (mean age, sex ratio, diagnosis, age of disease onset, recruitment setting); (iii) study design; (iv) conclusions and comments. Four authors independently extracted data for blind check of accuracy

### 4.4. Primary and Secondary Outcomes

The primary outcome of our study was the prevalence of misuse or diversion of prescribed stimulants among individuals with ADHD with prescribed stimulant therapy. As secondary outcomes, we considered characteristics associated with stimulant misuse/abuse or diversion.

We registered our review at OpenScienceFramework (OSF) with the doi identifier: DOI 10.17605/OSF.IO/DWB4Y, visualization link: https://osf.io/dwb4y/?view_only=da1e6576692d44b18a76d409c01a7121 (accessed on 11 August 2024).

## 5. Conclusions

Our systematic review highlights the complex interplay between ADHD, stimulant therapy, and the risk of misuse and diversion. As a whole, our findings are heterogeneous, indicating that while many individuals adhere to their prescribed medication regimens, there is variability in adherence, with occurrences of misuse behaviors. The variability of findings seems to be related to differences in the study design as well as the duration of the study period. The misuse of prescribed treatment for ADHD seems to be related to specific subject characteristics, including older age, previous or more frequent use of ADHD medication and previous diagnosis of alcohol/substance misuse, possibly indicating the need for tailored interventions addressing specific needs of this subject in order to achieve optimal treatment strategies without incoming misuse. To promote the correct use of stimulants, it is essential to develop age-specific interventions, such as targeted educational programs for older adults and prevention programs for adolescents and young adults, while conducting regular medication reviews and exploring non-pharmacological treatments to prevent tolerance. Integrated treatment plans should address both ADHD and SUD, with increased monitoring and support for individuals with a history of SUD. Preferably, extended-release formulations of stimulants should be prescribed to reduce misuse potential, and secure dispensing practices should be implemented. Additionally, training healthcare providers to recognize and prevent stimulant misuse, educating patients and their families about proper medication use, utilizing prescription-monitoring programs, and developing clear guidelines for safe prescribing are crucial steps in mitigating misuse risks and ensuring effective ADHD treatment. Despite the limited and heterogeneous evidence, by elucidating the prevalence and determinants of stimulant misuse, our findings contribute to the development of evidence-based strategies aimed at promoting the safe and appropriate use of ADHD medications while mitigating associated risks.

## Figures and Tables

**Table 1:** Summary of included studies divided by sample age in chronological order of publication.

Study	Population	Drugs	ADHD Assessment	Study Design	Prevalence and Modality of Stimulant Diversion and Abuse/Misuse	Conclusion and Comment
Adult sample						
Levin et al., 2006 [13]	Total sample of 98 methadone-maintained patients with ADHD (56♂, 42♀; age $\bar{x}$ = 39 ± 7) treated with Mph (N = 32, age $\bar{x}$ = 40 ± 6; start medication age $\bar{x}$ = 40 ± 6), bupropion and placebo. 53% of the sample had co-occurrent cocaine dependence/abuse	Mph sustained release	WURS, AARS and clinical interview	DB Plc-controlled 12-week clinical trial comparing the efficacy of Mph and bupropion to placebo on ADHD symptoms	No evidence that stimulant medication was diverted, misused or abused during 12-week study	Sustained-release Mph and sustained-release bupropion did not demonstrate a significant advantage over placebo in alleviating ADHD symptoms. The use of sustained-release Mph can be considered safe in terms of potential misuse, and there was no evidence of it worsening cocaine use
Wilens et al., 2006 [21]	Total sample of 98 medicated subjects (65♂, 33♀; age $\bar{x}$ = 20.8 ± 5.1), among them 55 individuals (45♂, 10♀; age $\bar{x}$ = 21.8 ± 5.7.; start medication age $\bar{x}$ = NA) reported current prescription for ADHD medication	Stimulant medication	DSM-III-R	Longitudinal 10 years case–control family study to evaluate the prevalence and correlates of ADHD stimulant diversion and misuse	Among the ADHD sample (N = 55), 11% reported selling medications and 22% reported misusing their medications taking excessive amounts or misuse it in order to “get high”. Individuals with conduct or SUD accounted for the misuse and diversion	Individuals with ADHD, especially those without conduct or SUD, use their medications responsibly. However, closely monitoring medication use and choosing medications with low risk of diversion or misuse in ADHD individuals with comorbid conduct or SUD is recommended
Darredeau et al., 2007 [17]	Total sample of 66 individuals (35♂, 31♀; age $\bar{x}$ = 22.3 ± 8.7; start medication age $\bar{x}$ = 21.6 ± 10.4) with prescribed ADHD medications	Mph	ADHD symptom checklist based on DSM-IV diagnostic criteria	Observational cross-sectional study to investigate adherence, diversion, and misuse of stimulant	44% of the of the total sample diverted their medication. Among them, 97% reported giving away their medication, 17% reported selling it, and 14% reported doing both. 29% of the total sample reported misusing their medications. Of those who reported ever misusing their medication, 84% reported oral misuse, 74% reported intranasal use, and 11% reported smoking it. None reported intravenous use. In addition, 68% of Mph misusers reported deliberately mixing Mph with alcohol and/or illicit substances	Noncompliance with medication, diversion, and misuse are prevalent and interconnected. A key distinguishing factor between individuals who misuse Mph and those who do not, seems to be their history of substance use. In this light, closely monitor prescriptions for these individuals are recommended.
Looby and Earleywine, 2009 [19]	Total sample of 157 individuals (44♂, 113♀; age $\bar{x}$ = 27.4 ± 8.9; start medication age NA) with ADHD, among them, 70.7% had prescribed ADHD medications	Mph	ASRS	Observational cross-sectional survey study exploring the role of positive and negative prescription stimulant-related expectancies in recreational and medical users of ADHD medication	Hierarchical cluster analysis identified two distinct groups among participants: medical users (72%) and recreational users (28%). Medical users were, on average, older and reported more frequent use of ADHD stimulant medication each month. Recreational users were more likely to report snorting their medication (34.09%) compared to medical users (7.08%). No significant differences were observed in the proportions of gender or ethnicity between the two groups	Positive expectancies, but not negative expectancies, predicted the frequency of use. Moreover, recreational users reported fewer positive and negative expectancies compared to medical users
Bejerot et al., 2010 [22]	Total sample of 133 individuals (71♂, 62♀; age NA; start medication age $\bar{x}$ = 31.1 ± 10.9) with ADHD diagnosis and prescribed stimulant medications	Mph and Amph	DSM-IV	Observational longitudinal (2 years) study to explore factors linked to treatment persistence and to document side effects and reasons for discontinuation	Drug abuse was not detected in this cohort during the 2 years study	Medications tend to maintain their effectiveness over the long term for adult ADHD, with mild side effects. Stimulants can be considered safe in terms of potential misuse; however, this result should be interpreted considering that individuals with comorbid alcohol and drug abuse were excluded
McRae-Clark et al., 2011 [14]	Total sample of 14 individuals (6♂, 8♀; age $\bar{x}$ = 33.9 ± 13.2; start medication age $\bar{x}$ = NA) with history of stimulant misuse, abuse, or dependence treated with transdermal Mph	Transdermal Mph	DSM-IV	An 8-week, open-label trial evaluated the effectiveness of the Mph transdermal system	No misuse of study medication was observed during 8-week study	Mph transdermal system may be effective in improving ADHD symptoms in adults who have a history of misusing stimulant medications. It seems that the drug was not misused in this study; however, this result might be influenced by the relatively short duration of the investigation
Lensing et al., 2013 [23]	Total sample of 159 individuals (84♂, 75♀; age $\bar{x}$ = 37.6 ± 11.1; start medication age NA) with ADHD diagnosis, among them 151 with prescribed ADHD medications	Mph and Amph	DSM-IV	Observational longitudinal (mean observation time was 4.5 years) study to explore the alignment between patient-reported and physician-reported treatment adherence and outcomes in adults with ADHD	In the cohort of individuals receiving ADHD treatment (N = 151), physicians indicated a manifestation of distrust regarding the usage of a dosage exceeding the prescribed amount in 8.6% of cases even though 82.1% of primary care physicians did not suspect misuse of prescribed medication. Within the subset of participants who discontinued pharmacotherapy for ADHD (N = 48), instances of misuse were reported as a causative factor in 14.6% of cases	The majority of primary care physicians did not harbor suspicions of stimulant medication misuse. A substantial consensus was observed between the physician’s lack of suspicion and patients’ reports of stimulant misuse, reaching a high agreement level of 91.7%
Ginsberg et al., 2015 [15]	Total sample of 25 prisoners (25♂, 0♀; age $\bar{x}$ = 33.6 ± 10.8; start medication age $\bar{x}$ = NA) with ADHD diagnosis and prescribed OROS- Mph	OROS-Mph	DSM-5	Observational longitudinal (47 weeks) open-label study, as extension of a 52-week DB Plc-controlled trial, to asses’ long-term effects of ADHD pharmacotherapy. During the 99-week trial, prisoners were still in jail. Among trial completers, 25 were prospectively followed up clinically for 1 year (24/25, 96% participated fully or in part) and 3 years (20/25, 80% participation) after trial. At the 3-year follow-up, 75% (15 out of 20) of the respondents had been released from prison	No misuse of ADHD medication or side abuse of other drugs was detected by repeated, mandatory urine toxicology throughout the study period	Improvements in symptoms and functioning observed during a 52-week trial of OROS-Mph in long-term prisoners with ADHD and concurrent psychopathology, including substance misuse, appeared to endure up to 3 years after the trial’s conclusion (to 4 years of treatment in total). At the 3-year follow-up, most participants were employed and had not relapsed into criminal behavior or substance misuse, indicating the potential long-term benefits of the treatment
Youth sample						
Gordon et al., 2004 [16]	Total sample of 162 adolescents in treatment for SUD (104♂, 58♀; age $\bar{x}$ = 17.1 ± 1.4), among them 55 individuals (37♂, 18♀; age $\bar{x}$ = NA; start medication age $\bar{x}$ = NA) reported a lifetime diagnosis of ADHD (31 with current ADHD and 24 with past ADHD). 45.5% (n = 25) of the patients who reported a current diagnosis of ADHD had been treated with a psychostimulant medication prior to admission	Mph and Amph	Structured interview for ADHD	Observational cross-sectional study to explore prevalence and characteristics of adolescent patients with comorbid ADHD and SUD	Among the ADHD sample (n = 55), 41.8 % reported a lifetime psychostimulant abuse; the prevalence of abusers among individuals with current ADHD and prescribed psychostimulant medication (n = 25) is not available. Moreover, 20 % of the patients with co-morbid ADHD attested to illicit diversion of psychostimulant medication by sale, barter, or gift to others	Around 34% of adolescents in SUD treatment reported a lifetime ADHD diagnosis. Nearly one-third of the total sample acknowledged psychostimulant abuse, with those co-diagnosed with ADHD significantly more prone (p= 0.003) to report a history of psychostimulant abuse. For this susceptible SUD/ADHD group, treatment should prioritize nonstimulant medications with low abuse potential over easily abused and diverted psychostimulants
Molinaet al., 2021 [20]	Total sample of 341 individuals (252♂, 89♀; age $\bar{x}$ = 15 ± 1.5; start medication age NA) with ADHD diagnosis and prescribed ADHD medications	Stimulant medication	DSM-V	Observational cross-sectional study, derived from pre-randomization baseline data from RCT of a stimulant diversion prevention workshop, characterizing the risk for stimulant diversion	The diversion rate was 1% among the total sample	While diversion was infrequent among adolescents treated in primary care settings, the risk seems to rise notably for older adolescents (*p* < 0.001). To enhance prevention effectiveness, it might be crucial to leverage existing psychosocial strengths and address stimulant-specific attitudes, behaviors, and social norms before the vulnerability to diversion escalates, especially in the later years of high school and into college
Mixed adult and youth sample						
Bright et al., 2008 [18]	Total sample of 545 individuals (344♂, 201♀; age 12–17 yrs 20.7%, 18–25 yrs 35.6%, 26–34 yrs 18.0%, 35–39 yr 6.6%, ≥ 40 yrs 16.9%, 2.2% not reported; start medication age 6–12 yrs 20.4%; 13–17 yrs 23.3%, 18–24 yrs 17.2%, ≥ 25 yrs 26.8%, 12.3% not reported) which included 486 (89.2%) subjects with ADHD and prescribed ADHD medications	Mph and Amph	AARS	Observational cross-sectional survey study evaluating the misuse of prescription and illicit stimulants among individuals undergoing ADHD treatment	Approximately 14.3% of the total sample engaged in the abuse of prescription stimulants. Among those who abused, 67.9% used a single stimulant, 21.4% used 2 stimulants, 4.8% used 3 stimulants, and 6.0% used 4 or more stimulants. Short-acting agents were abused by 79.8%, long-acting stimulants by 17.2%, both by 2.0% and 1% other not specified formulation.. The most commonly abused stimulants were mixed amphetamine salts (40.0%), mixed amphetamine salts extended release (14.2%), and Mph (15.0%). Crushing pills for inhalation/snorting (75.0%) was the predominant method of abuse, followed by crushing and injecting (6.3%), microwaving/melting to snort (6.3%), and other methods (12.5%). Additionally, 16.5% of the total shared their prescription ADHD medications, with friends (67.0%) and relatives (28.4%) being the most common recipients	Individuals treated in an ADHD clinic face elevated risks of abusing prescription and illicit stimulants. The agents most frequently abused are those with characteristics conducive to a rapid high. This implies that long-acting stimulant preparations, designed for ADHD treatment, might have lower abuse potential compared to short-acting formulations
Bjerkeli et al., 2018 [24]	Total sample of 56,922 individuals (36,243♂, 20,679♀; age range 6–79; start medication age NA) with a Mph prescription, among them 44,244 individuals had ADHD diagnosis (gender NA; age NA; start medication age NA)	Mph	ICD-10	Observational longitudinal (1 years) study to identify overuse of Mph and to investigate patterns of overuse in relation to sociodemographic and clinical characteristics. Data were obtained from Swedish national pharmacy dispensing data	7.6% of the total sample were categorized as over-users (defined as having above 150% of the maximum recommended dose during 365 days from the first prescription fill). Among the ADHD group (44,244 individuals) the prevalence of over-users was 8.42% with 3.81% of them having above 200% of dosage needed	The prevalence of overuse appears to be associated with a previous diagnosis of alcohol and drug misuse, higher age, and prior use of ADHD medication, suggesting a potential link between exposure time and overuse
Guerra et al., 2022 [25]	Total sample of 25,603 individuals (19,772♂, 5831♀; age $\bar{x}$ = 15.8 ± 11.9; start medication age NA) with a Mph prescription	Mph	Clinical	Observational longitudinal (13 years) study to assess the use of Mph and the extent of its abuse in the general population from 2005 to 2017 using a clustering classification method. Data were obtained from regional database Provence-alpes-côte d’Azur and Corsica health insurance	Over the 13 years under examination, the count of individuals receiving at least one dispensation of methylphenidate increased by 5.8 times. Within this group, the number of children rose by 5.2 times, whereas the count of adults surged tenfold. The clustering classification based on 4 quantitative active variables (number of prescribers, number of pharmacies, number of dispensing, quantity dispensed) calculated for each subject for the 9 months of follow-up for each year identified that 2.1% of the sample were “deviant users” and 97.9% were “no-deviant users”. Deviant group had older age, more frequent use of psychoactive drugs (benzodiazepine, morphine, opiate substitution treatment) and more Ritalin and less Concerta	Given the rise in individuals exhibiting “deviant” behavior, it is crucial to raise awareness within the medical community and among patients about the risk of methylphenidate abuse. The recent expansion of ADHD indications in adults and broader prescription conditions necessitate heightened vigilance.

Abbreviations: ♀, female; ♂, male; AARS, ADHD rating scale; ADHD, attention deficit/hyperactivity disorder; Amph, amphetamine, racemic amphetamine, amphetamine salts; ASRS, Adult ADHD Self Report Scale; DB, double-blind; DSM-5, Diagnostic and Statistical Manual of Mental Disorders, fifth edition; DSM-IV, Diagnostic and Statistical Manual of Mental Disorders, fourth edition; DSM-III-R, Diagnostic and Statistical Manual of Mental Disorders third edition, revised; ICD- 10, International Classification of Diseases tenth edition; Mph, methylphenidate; NA = not available; Plc = placebo; SUD, Substance use disorder; WURS = Wender Utah rating scale; $\bar{x}$ = mean; yr(s), year(s);

## Data Availability

Data is contained within the article.

## Supplementary Materials

The following supporting information can be downloaded at: https://www.mdpi.com/article/10.3390/ph17081076/s1, Table S1. Summary of reported outcomes among individuals with ADHD undergoing psychostimulant therapy.
