# Supplementary material for: Examining the Myth of Prescribed Stimulant Misuse among Individuals with Attention-Deficit/Hyperactivity Disorder: A Systematic Review
Source: Pharmaceuticals (Basel). 2024 Aug 16;17(8):1076. doi: 10.3390/ph17081076 (PMC11357389; doi:10.3390/ph17081076)
Supplement: Supplementary file 1 [file pharmaceuticals-17-01076-s001.zip › pharmaceuticals-3122957-supplementary.pdf]

pharmaceuticals

Table S1. Summary of reported outcomes among individuals with ADHD undergoing psychostimulant therapy.

Study	Stimulant Abuse/Misuse	Stimulant Diversion
	Adult sample	
Levin et al., 2006 [13]	None	None
Wilens et al., 2006 [21]	22%	11%
Darredeau et al., 2007 [17]	29%	44%
Looby and Earleywine, 2009 [19] [a]	28%	N/a
Bejerot et al., 2010 [22]	None	N/a
McRae-Clark et al., 2011 [14]	None	N/a
Lensing et al., 2013 [23] [b]	8.6%	N/a
Ginsberg et al., 2015 [15]	None	N/a
	Youth sample	
Gordon et al., 2004 [16] [c]	41.8%	20%
Molina et al., 2021 [20]	N/a	1%
	Mixed adult and youth sample	
Bright et al., 2008 [18] [d]	14.3%	16.5%
Bjerkeli et al., 2018 [24] [e]	8.42%	N/a
Guerra et al., 2022 [25]	2.1%	N/a

a: 29.3% of the sample was not undergoing stimulant therapy; b: 5.03% of the sample was not undergoing stimulant therapy; c: 54.5% of the sample was not undergoing stimulant therapy; d: 10.8% of the sample don't have an ADHD diagnosis or was not undergoing stimulant therapy, e: 22.5% of the sample don't have an ADHD diagnosis ; N/a: Not available.
